# Assessing knowledge of migrant sexual reproductive health and rights: a national cross-sectional survey among health professionals in Sweden

**DOI:** 10.3389/fsoc.2024.1356418

**Published:** 2024-05-30

**Authors:** Birgitta Essén, Ayanthi Wickramasinghe, Lise Eriksson, Irina Vartanova, Andrey Tibajev, Pontus Strimling

**Affiliations:** ^1^Department of Women’s and Children’s Health, Faculty of Medicine, Uppsala University, Uppsala, Sweden; ^2^Faculty of Social Sciences, Business and Economics, and Law, Åbo Akademi University, Turku, Finland; ^3^Institute for Future Studies, Stockholm, Sweden

**Keywords:** migrants, sexual reproductive health and rights, migrants healthcare, health professionals, healthcare providers, norms and values, sexual and reproductive healthcare

## Abstract

**Introduction:**

Despite the commitment of the Swedish government to ensuring equal access to Sexual Reproductive Health and Rights services for all citizens, shortcomings persist among the migrant population. In cases where healthcare providers lack sufficient knowledge or hold misconceptions and biases about these contentious issues, it can lead to the delivery of suboptimal care. Therefore, the objective of this study was to assess the level of knowledge of Swedish healthcare providers on global and Swedish migrant Sexual Reproductive Health and Rights.

**Methods:**

A national cross-sectional study was conducted using a questionnaire consisting of seven questions related to global and Swedish migrant Sexual Reproductive Health and Rights. The questionnaire was distributed among midwives, nurses, gynecologists and obstetricians, and hospital social workers (*N* = 731). The analysis was guided by the Factfulness framework developed by Hans Rosling to identify disparities between healthcare providers’ viewpoints and evidence-based knowledge.

**Results:**

There was an overall lack of knowledge among the health care providers on these issues. The highest correct responses were on the question on abandonment of female genital cutting/mutilation after migration (74%). The findings indicated that healthcare providers originating from Sweden, physicians, those with fewer years of clinical experience, and exhibiting more migrant-friendly attitudes, demonstrated a higher level of knowledge regarding global and Swedish migrant Sexual and Reproductive Health and Rights.

**Conclusion:**

This study demonstrates that healthcare providers lacked knowledge of global and Swedish migrant Sexual Reproductive Health and Rights, which was almost uniformly distributed, except among those with more comprehensive and recent education. Contrary to expectations, healthcare professionals did not primarily rely on their education and experiences but were influenced by their personal values and opinions. The study underscores the importance of upgrading knowledge in Sexual Reproductive Health and Rights and encourages policymakers, professionals, and students to base their opinions on well-founded facts, particularly in the context of a diverse and globalized society.

## 1 Introduction

Over the past decades, there has been a notable surge in the global population of international migrants, refugees, and internally displaced individuals. Several European countries, including Sweden, have witnessed an high influx of migrants (Byström and Frohnert, 2017; World Health Organization, Regional Office for Europe, 2018; ‘International Migration 2020 Highlights, 2020). According to the United Nations (UN) data as of 2020, Sweden is home to 2 million migrants, constituting 17% of the total population (Global Migration Data Analysis Centre and IOM UN Migration, 2020; International Migration 2020 Highlights, 2020). The most substantial increases in immigration have originated from countries such as Iraq, the Former Yugoslavian Republic, Poland, Afghanistan, Eritrea, Syria, and Somalia during recent decades (Statistics Sweden, 2022).

Despite significant advancements made by countries, there remains a persistent shortfall in the provision of Sexual Reproductive Health and Rights (SRHR) services (Sustainable Development Goals | United Nations Development Programme, 2015). While research on all aspects of SRHR within migrant populations is lacking (Gagnon and Redden, 2016), there are several studies on maternal health, abortion, and contraception (Rodriguez-Alvarez et al., 2016; Emtell Iwarsson et al., 2019; Heino et al., 2020). International reports (Gagnon and Redden, 2016; World Health Organization, Regional Office for Europe, 2018) and studies in Sweden (Essén et al., 2002; Esscher et al., 2013) have highlighted suboptimal maternal health outcomes among specific groups of migrant women, particularly those originating from lower-income countries, when compared to their non-migrant counterparts. Research indicates a higher incidence of induced abortions and lower rates of contraceptive counseling and contraceptive use among migrant women (Rodriguez-Alvarez et al., 2016; Emtell Iwarsson et al., 2019; Heino et al., 2020). To improve migrant SRHR, it is imperative to overcome the multifaceted barriers entrenched in factors such as ignorance, inequality, legal frameworks, policies, social norms, and values. These barriers collectively hinder individuals from accessing optimal sexual reproductive healthcare (SRHC).

Sweden is recognized as a leader in gender equality, with exceptionally liberal and egalitarian values concerning issues of equity and reproductive rights (Arousell et al., 2017; World Values Survey organization, 2017). Despite Sweden’s commitment to multiculturalism ideals (Borevi, 2013; Wickström, 2015) and interventions facilitating migrant assimilation, there persist shortcomings in the health sector (Norredam et al., 2007), especially in SRHR. Shortcomings in migrant SRHR have also been demonstrated in other western countries (Gagnon and Redden, 2016; van den Akker and van Roosmalen, 2016). These deficiencies may stem from the distinctive demands of multicultural healthcare encounters, necessitating specific knowledge about migrants and migrant health (Binder et al., 2012). Inadequate knowledge, misinterpretations, and biases, have been recognized as additional factors contributing to suboptimal care provision (Essén et al., 2002; Esscher et al., 2013; World Health Organization, Regional Office for Europe, 2018). Healthcare providers (HCPs) have, at times, resorted to generalizations or invoked a patient’s cultural background as an explanatory factor in the context of Swedish healthcare, due to a lack of evidence-based knowledge or the presence of certain prejudices and biases (Byrskog et al., 2015; Rothlind et al., 2018). Additionally, the personal values upheld by HCPs, influenced by societal norms, significantly shape healthcare interactions with migrants (Eriksson et al., 2022).

The Swedish government has remained unwavering in its commitment to delivering culturally sensitive care and integrating an equality perspective into SRHR services for all citizens, including the substantial migrant population (Socialstyrelsen, 2015). Despite these efforts, a significant disparity persists among migrants from Low-Income Countries in various aspects of SRHR. One of the primary barriers to achieving equitable SRHC services is the absence of evidence-based knowledge and the presence of misconceptions among HCPs when engaging with foreign-born patients (Essén et al., 2002). This is particularly evident in SRHR areas such as sexuality, reproduction, and female genital cutting/mutilation (FGC/M), which are often controversial topics and sources of cross-cultural conflicts due to pre-existing biases and struggles between private and professional values (Ramondetta et al., 2011; Arousell and Carlbom, 2016; Fiala et al., 2016; Eriksson et al., 2022). Although healthcare policies and guidelines have been formulated to equip medical professionals with essential knowledge and tools (World Health Organization, Regional Office for Europe, 2018), the information can sometimes be conflicting and challenging to apply at an individual level. Moreover, the confluence of rapid global developments, shifting migration patterns, public discourse surrounding contentious SRHR-related topics, and the need to maintain an evidence-based and up-to-date understanding of global and Swedish SRHR can be daunting. This deficit in evidence-based knowledge can have adverse effects on clinical consultations, fostering misunderstandings and eroding trust between HCPs and migrant patients, resulting in suboptimal care.

Ideologies and values strongly influence the inherently politicized field of SRHR. The Guttmacher-Lancet Commission defines SRHR as, the right of individuals and couples to decide freely and responsibly the number, spacing, and timing of their children, to have the information and means to do so, and to have the highest attainable standard of sexual and reproductive health, free from discrimination, coercion, and violence. This definition encompasses a broad range of issues including access to contraception, safe abortion, maternal healthcare, prevention and treatment of sexually transmitted infections (STIs), and comprehensive sexuality education (Starrs et al., 2018). This is consistent with Swedish policy (Socialstyrelsen, 2015). However, the UN has concurrently instituted a distinct policy strongly emphasizing religious freedom and cultural sensitivity (OHCHR, 1992), potentially creating a conflict between these policies. On the other hand, grounding beliefs on facts and empirical data present a challenge in SRHR as they are often influenced by personal values, prejudices, and driven by ideology (Nyström et al., 2022). These factors can pose challenges in multicultural healthcare settings. Therefore, this study aimed to assess the level of knowledge and fact-based perspectives of Swedish HCPs on global and Swedish migrant SRHR, in order to understand the scope of the challenge and identify effective strategies for overcoming it. Furthermore, we sought to investigate potential connections between HCPs’ empirical knowledge and their demographic attributes, including gender, educational background, country of origin, years of clinical experience, and social values. HCPs social values evaluated from the opinions derived from three key questions, which assess respondents’ openness to migrants from diverse backgrounds, their views on migrants cultural impact, and their perception of migrants’ overall contribution to Swedish societal well-being.

### 1.1 Explanatory model and conceptual framework

Our explanatory model and conceptual framework is inspired by the “Factfulness” framework developed by Rosling (2018). Rosling advocates for individuals to rely on robust factual information and cultivate an evidence-based approach to thinking. He provides a pragmatic framework comprising 10 instincts aimed at combating our inclination toward biases and misconceptions, ultimately enabling us to utilize data for clearer insights. These instincts are: the Gap instinct, Negativity instinct, Straight Line instinct, Fear instinct, Size instinct, Generalization instinct, Destiny instinct, Single Perspective instinct, Blame instinct, and the Urgency instinct. Factfulness involves counteracting ignorance by supporting fact-based claims about global trends in health and welfare, thereby promoting a fact-based worldview. These instincts help individuals recognize and rectify potential prejudices. Some of these instincts are also applicable to the context of multicultural SRHC encounters (Essén and Mosselmans, 2021). In alignment with this framework, we administered seven fact-based questions on SRHR to gauge misconceptions among HCPs. This approach allowed us to identify any disparities between HCPs perspectives and evidence-based knowledge.

## 2 Materials and methods

### 2.1 Study design and data collection

A cross-sectional survey was conducted targeting midwives, nurses, gynecologists and obstetricians, and hospital social workers working in SRHC across all regions of Sweden. This encompassed outpatient and inpatient care in gynecology, obstetrics, and reproductive health [International Maternal and Reproductive Health and Migration (IMHM), 2022].

The survey is a part of a larger project investigating how norms, values, and knowledge impact the interaction between HCPs and migrant patients (Eriksson et al., 2022). Data were collected between January and May 2021 by distributing online questionnaires through the Swedish Society for Obstetrics and Gynecology email list for members and to HCPs through emails distributed by gatekeepers, such as heads of department and midwifes at public and private clinics in Sweden. Participants could answer the questionnaire without exclusion based on workplace or profession, resulting in a total non-probability sample of 1,257 respondents.

The questionnaire underwent two rounds of pilot testing, initially with 20 HCPs followed by qualitative interviews, and subsequently with 200 respondents from the general public. It included questions on demographics and work characteristics, moral issues related to SRHR, gender equality, migration, and seven questions on global and Swedish migrant SRHR. The questions on moral issues were predominantly derived from the World Values Survey and the European Social Survey (Eriksson et al., 2022). The knowledge questions, the focus of this paper, had three possible alternatives with one correct response.

To gain an understanding of HCPs’ perceptions of SRHC outcomes in both a global and migrational context in Sweden, we employed seven fact-based questions. Four of these questions pertained to maternal mortality (#1,4) and family planning, with a focus on accessibility to contraceptive counseling (#2,3). These questions encompassed global maternal mortality rates (World Health Organization et al., 2015), maternal mortality among migrants within Sweden (Esscher et al., 2013), global access to contraception (Starrs and Anderson, 2016), and the mean age of marriage for women in the Middle Eastern and North African (MENA) region (UNICEF, 2018). These questions were strategically framed to encapsulate the evolution of UN sexual reproductive health initiatives from the 1980s, such as the Millennium Development Goals (MDGs), and the ongoing Sustainable Development Goals (SDGs). The following two questions explored social determinants affecting human reproduction and cultural shifts in harmful practices (#5,6). Specifically, they examined how social determinants determine the number of children, overshadowing considerations of religion or culture (Rosling, 2018). These questions assessed awareness of the abandonment of FGC/M practices after migration from regions with high FGC/M prevalence, such as Somalia, Eritrea, and Ethiopia in the Horn of Africa, by individuals relocating to zero-tolerance FGC/M areas (Johnsdotter and Essén, 2016). Our final aim was to determine HCPs awareness of the organization and financing of culturally and religiously sensitive abortion counseling in Swedish hospitals (#7)—an area marked by paradoxes. This explores knowledge of the current paradox in Swedish policy, which advocates for not only professional but also religious advisors/counselors within SRHC. The funding of religious abortion counseling is split between both the government and religious communities. Certain religious leaders advise women not to undergo abortions, which is a conflict with the Swedish policy that women have a right to decide (Swedish Government, 2018; Arousell et al., 2019) (Table 1).

**Table 1:** The seven fact-based questions asked from 731 HCPs in Sweden in 2021.

**#1**	**Global maternal mortality** In the last 25 years, the global maternal mortality rate (per 100,000 live births)… Has remained unchanged Increased by 25% Decreased by 50%*
**#2**	**Access to contraception** How many women of reproductive age in the world currently have access to modern contraceptives? 40% 60% 80%^*^
**#3**	**Marriage age MENA** The average age for women to get married in Sweden is 34 years. What is the corresponding age for women in the Middle East and North Africa? 18 years 24 years^*^ 28 years
**#4**	**Migrants’ maternal mortality** Approximately, how much higher is the risk of maternal mortality for migrant women from low-income countries (e.g., Somalia) compared to Swedish-born women in Sweden? Two times higher Four times higher Six times higher^*^
**#5**	**Children per woman** Which factor do we know is most strongly associated with the number of children a woman gives birth to in her lifetime? Family’s religion Family’s culture Family’s income^*^
**#6**	**Attitudes to FGC** In Sweden, many migrants come from countries where both male and female circumcision is practiced. How do their attitudes toward female circumcision change when migrant families live in Sweden? They become more permissive They become less permissive^*^ Their permissiveness does not change
**#7**	**Religious counseling** Patients in Sweden can receive counseling on contraceptives and abortion from religious advisors at hospitals. Are their activities funded by: Only the state Only religious organizations Both the state and religious organizations^*^

^*^Correct response.

### 2.2 Statistical analysis

To construct the sample used in the analyses, we first excluded 216 respondents who did not fit the target population, such as those working in fields of medicine other than SRHC, in different professions, or who were retired (above the age of 67). An additional 310 respondents were excluded based on a list-wise deletion due to missing values on the used variables. The analytical sample consisted of 731 HCPs. Our outcome variable, a fact-based knowledge of global and Swedish migrant SRHR, is the number of correct responses on the knowledge questions, ranging from 0 to 7. These questions were at the very end of the survey and caused most of the case-wise deletion due to respondents choosing to opt out of the survey before completion. We used descriptive statistics to demonstrate the distribution of the number of correct responses among the respondents and the proportion of correct responses per knowledge question. Ordinary Least Squares (OLS) regression models were employed to assess the average differences in knowledge between HCPs across the categories of gender, education, origin country, and number of clinical years. Finally, we analyzed the connection between opinions about migrants and knowledge. This was done by including an index of opinions based on three questions in the survey. The questions were: to what extent the respondents think Sweden should allow migrants of different races or ethnic groups to come and live here, if Sweden’s cultural life was undermined or enriched by migrants, and if migrants make Sweden a worse or better place to live. The index theoretically ranges from 0 (lowest possible response on all three questions) to 1 (highest possible response to all three questions). The three questions were taken from the European Social Survey and had an alpha score of 0.79.

### 2.3 Ethical approval

Ethical approval was granted by the Regional Ethical Committee (Dnr 2018/425) and by the Swedish Ethical Review Authority (Dnr 2020-07187). Informed written consent was obtained prior to the distribution of the questionnaires. All respondents were anonymous, and no identifying data were collected.

## 3 Results

The demographics and professional characteristics, as well the index on opinions about migrants, of the HCPs included in the analysis are presented in Table 2.

**Table 2:** Descriptive statistics of 731 HCPs in Sweden in 2021.

Variable	Range	Mean	SD
Gender			
Male	0/1	0.08	
Female	0/1	0.92	
Origin country			
Sweden	0/1	0.88	
Rest of the world	0/1	0.12	
Education			
Midwife/nurse	0/1	0.55	
Physician	0/1	0.42	
Hospital social worker	0/1	0.03	
Clinical years	0–50	17.75	11.46
Opinions about migrants	0.11–1.	0.80	0.18

*N* = 731. Categorical variables coded 0/1.

**Figure 1:**
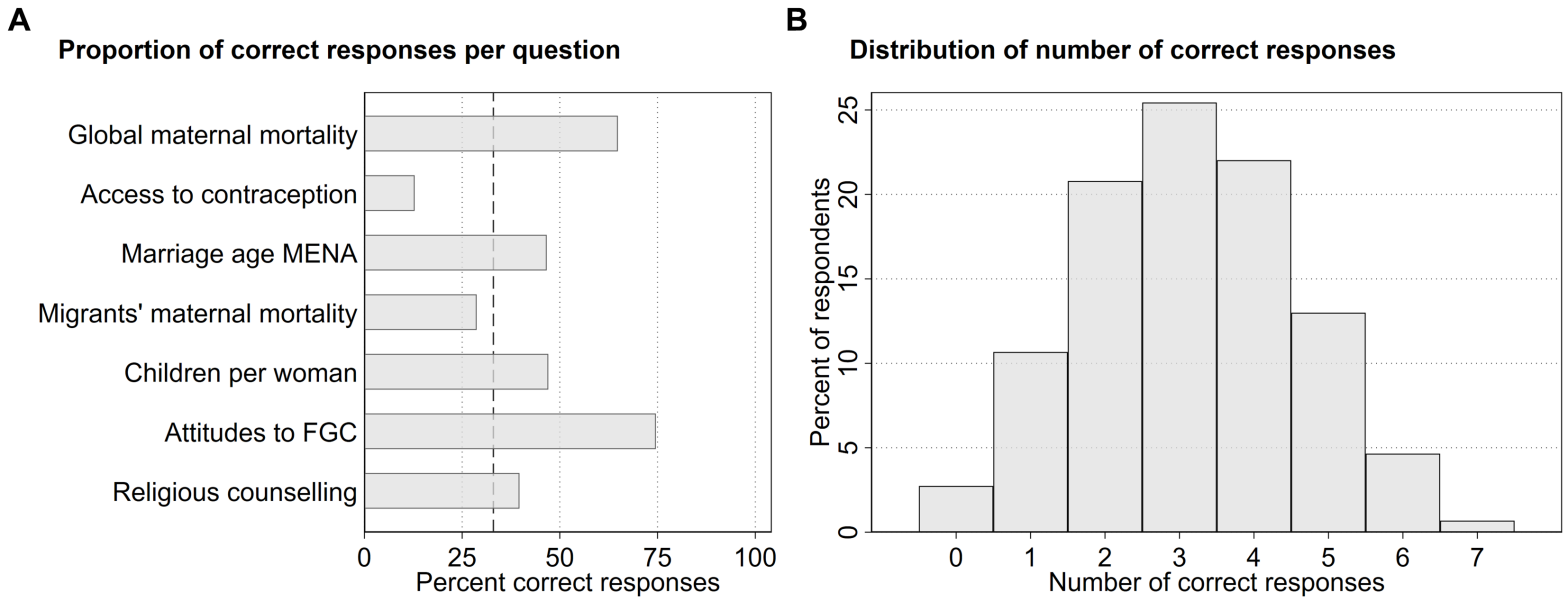
Descriptive statistics of knowledge questions from 731 HCPs in Sweden in 2021.

Figure 1A describes the proportion of correct responses per question, while Figure 1B shows the distribution of correct responses to the surveyed questions. On average, random responses would yield approximately 0.33 correct answers per question, which is represented by a dashed line in Figure 1A. The highest percentage of correct responses was for “Attitudes to FGC/M after Migration,” with a 75% accuracy rate. Conversely, the lowest percentage of correct responses, 13%, was for the question on “Access to contraception globally.” Across all questions and the entire sample, HCPs provided the correct answer on average 45 percent of the time, translating to 3.14 correct answers (SD = 1.46). As visible in Figure 1B, the overall distribution of the number of correct answers is approximately normally distributed.

### 3.1 Correlation with HCPs demographic and work characteristics

**Figure 2:**
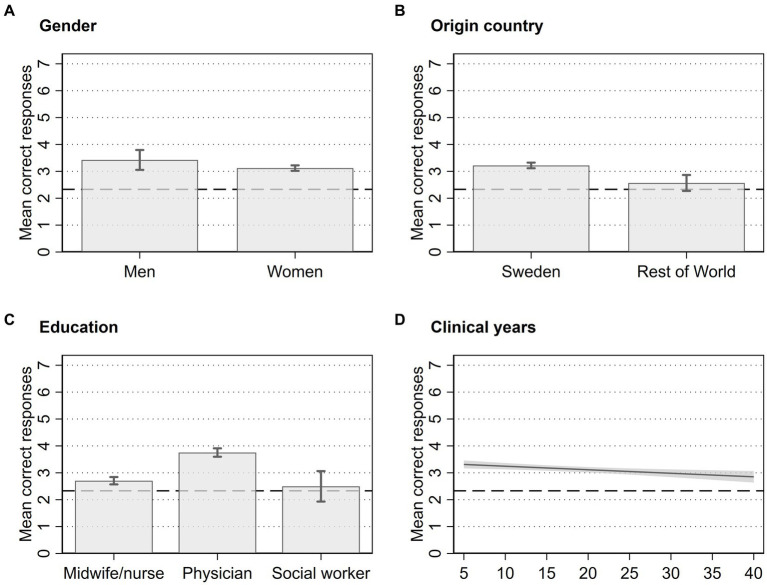
Association between number of correct responses and respondent characteristics of 731 HCPs in Sweden in 2021. Error bars represent 95% CI from regression.

Figure 2 displays the predicted mean correct responses after a multivariate OLS regression. The full results are presented in Supplementary Table S1. Predictions were made for each of the included categories, such as gender, origin country, education, and clinical years, with average values for the other included categories. The dashed line indicates the expected number of correct responses if the questions were answered at random, equal to 2.33.

We identified several statistical associations between the demographic and professional characteristics of HCPs and the number of correct responses. Specifically, HCPs from Sweden scored higher than those from the rest of the world (Figure 2B), physicians scored higher than midwives/nurses and social workers (Figure 2C), and HCPs with fewer clinical years scored higher than those with more clinical years (Figure 2D). However, our data did not reveal an association between gender and the number of correct responses (Figure 2A). Additionally, we checked for associations with region or the type of clinic where HCPs worked, but these did not yield significant results (not shown here).

### 3.2 Correlation between knowledge and opinions about migrants

**Figure 3:**
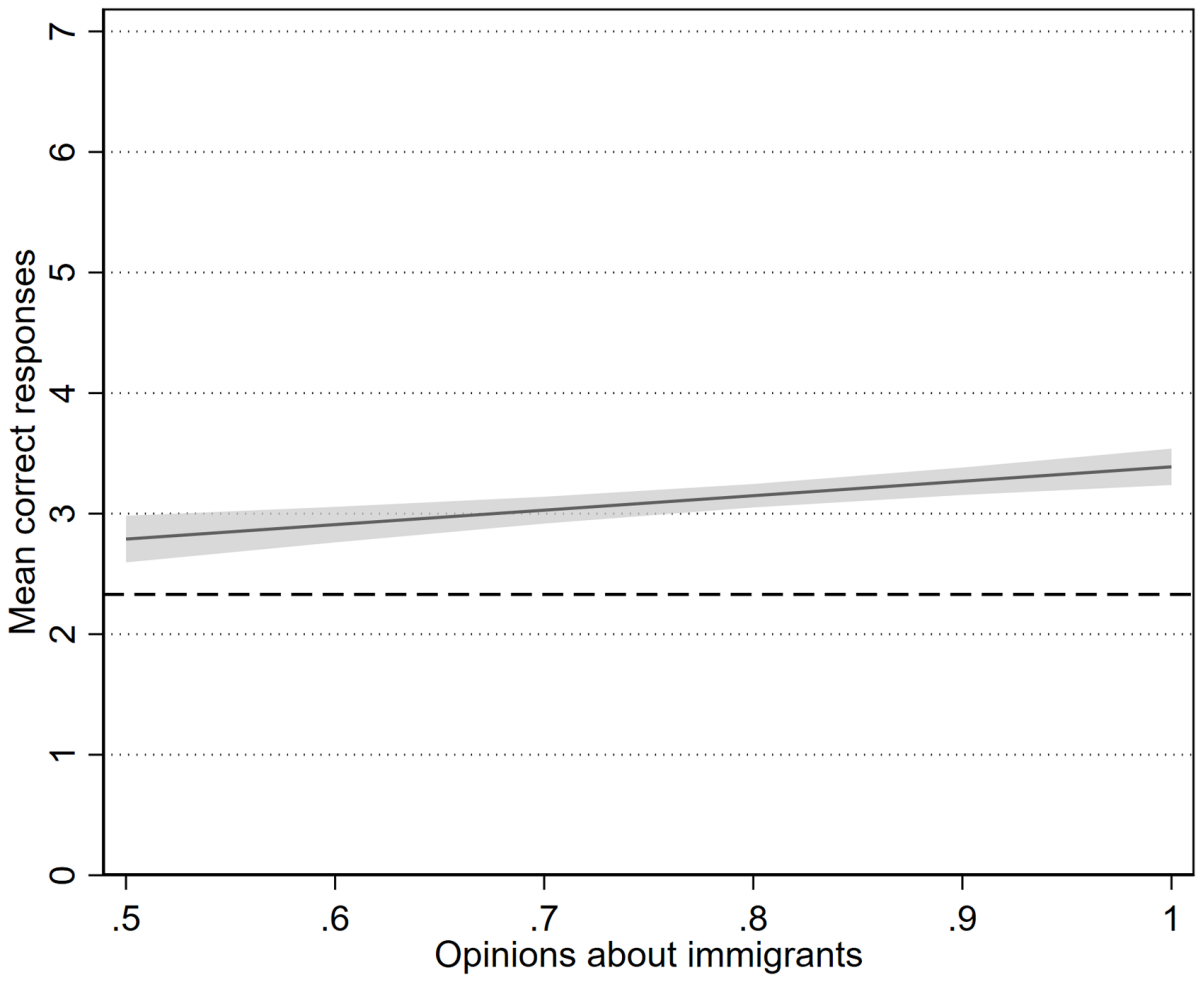
Association between number of correct responses and opinions about migrants from 731 HCPs in Sweden in 2021. Shaded area represents 95% CI from regression.

As the final step in the analysis, we examined the connection between opinions about migrants and knowledge. We added the index on migrants to the regression model, with higher values indicating more migrant-friendly opinions. As the result in Figure 3 displays (full results in Supplementary Table S1), there is a positive correlation between more migrant-friendly opinions and a higher score on the knowledge questions. HCPs with the highest value on opinions about migrants have approximately 0.6 more correct answers than the HCPs who answered in the middle on the three questions about opinions about migrants. The dashed line indicates the expected number of correct responses if the questions were answered at random, equal to 2.33.

## 4 Discussion

This study adopts a novel approach in SRHR from a global and migrational perspective within Sweden. Notably, no existing studies have undertaken a quantitative assessment of HCPs knowledge concerning SRHR, which could be linked to the suboptimal care received by migrants (Essén et al., 2002; Esscher et al., 2013). Interestingly, the HCPs in our study demonstrated a low level of global and Swedish migrant SRHR knowledge, which was not much higher than random responses in question #2 and #4, although they performed much better in the rest of the questions. The findings additionally indicated that HCPs who originated from Sweden, were physicians, had fewer clinical years, and had more migrant-friendly attitudes possessed more knowledge on global and Swedish migrant SRHR. While the Factfulness framework has been used to evaluate the management of FGC/M at a policy level (Essén and Mosselmans, 2021), our study uniquely extends this evaluation to the individual level. This study demonstrates the need for HCPs to acquire and maintain a fact-based worldview within migrant SRHR, which can, in turn, lead to improvements in SRHC.

The knowledge of HCPs regarding global patterns of maternal mortality appears to be quite robust. When a similar question was posed by Rosling (2018) to a random sample of the Swedish population, less than one-third believed that global extreme poverty was in decline, performing worse than random chance. This aligns with the “negativity instinct” of the Factfulness framework. Rosling claims that there is a human tendency to assume that things are generally worsening. To counteract this instinct, individuals should focus on trends rather than just the absolute values of measures, while keeping in mind that positive developments are often overlooked because they are not considered newsworthy. The fact that our participants overcame this instinct could be attributed to the influence of education. This outcome also highlights the significant role played by the MDGs and other 21st-century global development agendas in reducing maternal mortality.

The question concerning access to modern contraceptives globally received the fewest correct answers. Even among HCPs, it seems that a prejudice exists in assuming that conservative family values, emphasizing the unacceptability of premarital sex, lead to limited usage and availability of modern contraceptives in certain countries. This could be linked to the “gap instinct” described in the Factfulness framework (Rosling, 2018), where there is often a tendency toward binary thinking, seeing things in terms of black or white, yes or no, right or wrong. Rosling supports this by emphasizing how grouping things into distinct and often opposing categories and assessing countries based on their income and other factors create a perceived gap between them. However, he suggests that a more precise viewpoint would consider economic development as a spectrum, with a considerable middle ground, particularly visible in the so-called middle-income countries. This may offer a plausible explanation for the lower scores obtained by HCPs on this question.

The question on the mean age of marriage in MENA countries was answered correctly by almost half of the participants, while the question determining the number of children saw a moderate number of correct answers. These questions were chosen as there are many misconceptions and prejudices globally surrounding migrants from this area (Arousell and Carlbom, 2016). To answer this question, knowledge of socioeconomic and sociocultural changes is also necessary. The prevalence of child marriages in these regions is on the decline (UNICEF, 2018), challenging HCPs to keep up and maintain a fact-based worldview. The prejudice held toward women from the MENA region may be associated with the “destiny instinct” (Rosling, 2018). The destiny instinct suggests that inherent traits dictate the fates of individuals, nations, religions, or cultures. Similarly, HCPs beliefs on the number of children can be explained by assigning the trait of fecundity to some religions and cultures and the aforementioned “destiny instinct” by assuming that having many children is a product of unchangeable factors. While in reality, the main determinant for the number of children per woman depends on socioeconomic factors such as income (Lesthaeghe, 2010). To prevent such biases as the destiny instinct, focusing on gradual advancements, regularly updating their knowledge, and seeking out instances of cultural transformation are essential.

The second lowest correct responses were to the question about the risk of maternal mortality for migrants from Low-Income Countries. Although it has been known since the 90s that maternal mortality rates among migrant women are high not only in Sweden and other Nordic countries (Esscher et al., 2013; Wahlberg et al., 2013; Vangen et al., 2017) but also in other European countries, the question can be considered difficult as it only suggests options acknowledging an increased odds ratio of maternal mortality for this group. HCPs being unaware of how high the risk migrant women from Sub-Saharan Africa face during childbirth could also be a cause for suboptimal care and a further increase in migrant maternal mortality.

The question regarding the widespread abandonment of FGC/M after migration received the highest number of correct answers. This question aimed to gauge whether HCPs have grasped the evolving understanding regarding the cessation of this practice, particularly since this evidence-based knowledge is not well-reflected in current FGC/M policy documents (Essén and Mosselmans, 2021). Research shows a cultural shift toward abandoning FGC/M due to transformed attitudes post-migration (Johnsdotter and Essén, 2016; Wahlberg et al., 2017). These findings are also acknowledged by WHO in Europe (World Health Organization, Regional Office for Europe, 2018) and the National Board of Health and Welfare in educational materials targeting HCPs. Additionally, studies (Johnsdotter and Essén, 2016) indicate that FGC/M is rarely encountered in Western settings, and HCPs rarely encounter recently performed cases. The personal experiences and knowledge of HCPs regarding this cultural change could have helped counter the “destiny instinct” mentioned above and the “Straight line instinct” within the Factfulness framework (Rosling, 2018; Essén and Mosselmans, 2021), and hence providing insights into where the trajectory had bent. According to the “Straight line instinct,” we possess a natural capability to project along linear trend lines and excel at recognizing trends and extending them linearly, such as presuming that population growth will follow the same path as recent historical data. However, genuine linear behavior is relatively rare. Frequently, trends encounter limits and start to level off or follow a pattern of rise and subsequent decline. Therefore, adopting a fact-based approach entails refraining from assuming that trends will invariably follow a linear path.

Respondents barely outperformed random chance with a 39% correct response rate to the last question concerning policy and funding for religiously affiliated abortion counseling. This question aimed to explore precise knowledge regarding the paradoxes between Swedish policy, law, and clinical practice. The funding of religious abortion counseling is shared between the government and religious communities, excluding the Church of Sweden, which uses its own resources (The Swedish Agency for Support to Faith Communities, SST (2021), 2017). This serves a dual purpose: promoting and safeguarding gender equality and a woman’s autonomy in making SRHR decisions (Swedish Government, 2005). However, it also necessitates HCPs to respect a diversity of cultural and religious values (Swedish Government, 2005; Socialstyrelsen, 2015), some of which may endorse gender inequality and view women’s SRHR as a collective family matter rather than individual decisions (Arousell et al., 2017). This complexity arises from religious leaders advising against abortions, conflicting with Swedish policy (Swedish Government, 2018) that upholds women’s right to decide (Arousell et al., 2019). Multiculturalist healthcare policies sometimes preserve cultural practices that simultaneously threaten women’s reproductive rights and freedoms. In other instances, these policies reinforce the perception of huge cultural differences, contributing to generalizing patients from migrant groups. This may hinder HCPs effective communication with individual patients from these backgrounds (Johnsdotter and Essén, 2016). The shared financing of counseling between the government and religious communities appears inconsistent with the laws and guidelines defining the mission of HCPs. Understanding these policy inconsistencies related to gender and cultural diversity in Swedish reproductive health and SRHR could assist HCPs in maintaining a balance between adhering to healthcare system values and being receptive to potential conflicting values in individual encounters in a multicultural healthcare setting.

The country of origin of the HCPs revealed that respondents from Sweden scored higher than those from other countries. Sweden boasts a well-developed educational system with a strong emphasis on SRHR and migrational research (Swedish Government, 2022). This could have provided native Swedish HCPs with an advantage. Additionally, Swedish culture is generally more open and accepting of discussions surrounding SRHR, potentially making participants born in Sweden feel more comfortable and knowledgeable on the subject.

In our study, physicians demonstrated more knowledge of global and Swedish SRHR than other HCPs. Several factors could contribute to this disparity. Physicians typically receive comprehensive medical education with extensive training in global health and reproductive health, potentially providing them with an advantage over their counterparts who are nurses/midwives and hospital social workers. Physicians may also gain more insight and knowledge from their experiences gained through their clinical encounters with migrant patients.

The analysis also indicated that HCPs with fewer clinical years scored higher than those with more clinical experience. Junior HCPs better knowledge and awareness of SRHR may be attributed to their recent completion of medical education. Medical education has evolved over time, with more recent graduates receiving more comprehensive global health education and reproductive health training. In contrast, senior HCPs may have a more skewed perception of the world’s situation, potentially believing it to be worse than it actually is, as suggested by Rosling (2018).

In our extended analysis, we explored the association between knowledge and opinions about migrants among HCPs. A higher level of knowledge on global and migrant SRHR was demonstrated by HCPs with migrant-friendly opinions. This finding suggests that the knowledge possessed by HCPs in the field of SRHR is influenced by their opinions of migrants, which could be based on personal values. Therefore, HCPs knowledge is not only grounded in what they have learned through formal education, research, and readings, as well as their practical experiences, but is also rooted in their personal values and moral beliefs (Eriksson et al., 2022; Tibajev et al., 2022).

### 4.1 Strengths and limitations

Our study possesses several notable strengths. Firstly, it contains a robust sample size, including 1,041 HCPs working within Swedish reproductive health, representing all regions across Sweden. Another strength of the study was that all healthcare providers were included in this study and not only physicians, as in most studies. The substantial sample size enhances the study’s statistical power and lends credibility to its findings, although the sampling method is a limitation as we cannot make statistical inference to the whole relevant population. Given the cross-sectional nature of our study, we cannot make claims of causality. There are other possible questions on knowledge that could be more comprehensive, and including a few questions is also a limitation of this study.

## 5 Conclusion

The findings of our study illuminate the overall knowledge held by HCPs and reveal certain misconceptions they harbor regarding global and Swedish migrant SRHR. Therefore, a key revelation was the relatively low knowledge level among HCPs in this field. HCPs with Swedish origin may have an advantage owing to Sweden’s educational system and its vested interest in migrant SRHR. Physicians outperformed other HCP groups, potentially attributed to the comprehensive medical and global health education they receive. Junior HCPs exhibited greater knowledge compared to their more experienced counterparts, possibly reflecting the evolution of global health education that incorporates agendas like the MDGs and SDGs. The study suggests that HCPs’ global and Swedish migrant SRHR knowledge is not solely derived from formal education and practical experiences but is deeply intertwined with their personal values and moral convictions. This aligns with the Factfulness framework, which addresses common biases hindering knowledge acquisition based on facts. These findings underscore the significance of enhancing knowledge among HCPs regarding migrants and SRHR. Such knowledge is paramount for delivering equitable and high-quality SRHR services to all individuals.

## Data availability statement

The raw data supporting the conclusions of this article will be made available by the authors, without undue reservation.

## Ethics statement

The studies involving humans were approved by Swedish Ethical Review Authority. The studies were conducted in accordance with the local legislation and institutional requirements. Written informed consent for participation in this study was provided by the participants’ legal guardians/next of kin.

## Author contributions

BE: Writing – review & editing. AW: Writing – original draft, Writing – review & editing. LE: Writing – review & editing. IV: Writing – review & editing. AT: Writing – review & editing. PS: Writing – review & editing.
