# Supplementary material for: Assessing knowledge of migrant sexual reproductive health and rights: a national cross-sectional survey among health professionals in Sweden
Source: Front Sociol. 2024 May 30;9:1356418. doi: 10.3389/fsoc.2024.1356418 (PMC11169828; doi:10.3389/fsoc.2024.1356418)
Supplement: Supplementary file 1 [file Table_1.DOCX]

Supplementary Material

**Table S1.** Regression models between number of correct responses and respondent characteristics of 731 HCPs in Sweden in 2021

|  | **Model for Figure 2** | **Model for Figure 3** |
| --- | --- | --- |
| Gender |  |  |
| Male | Ref. | Ref. |
| Female | -0.30 | -0.34 |
|  | (0.20) | (0.19) |
| Origin |  |  |
| Sweden | Ref. | Ref. |
| Rest of the world | -0.65*** | -0.61*** |
|  | (0.16) | (0.16) |
| Education |  |  |
| Midwife/nurse | Ref. | Ref. |
| Physician | 1.05*** | 1.01*** |
|  | (0.11) | (0.11) |
| Hospital social worker | -0.21 | -0.25 |
|  | (0.30) | (0.29) |
| Clinical years | -0.01** | -0.01* |
|  | (0.00) | (0.00) |
| Opinions about migrants |  | 1.20*** |
|  |  | (0.29) |
| Constant | 3.29*** | 2.33*** |
|  | (0.22) | (0.32) |
| Adj-R2 | 0.1431 | 0.1619 |
| N | 731 | 731 |

Dependent variable number of correct responses (1-7). Standard errors in parentheses.

*p<0.05; **p<0.01; ***p<0.001
